# The effect of intermittent fasting on the clinical and hematological parameters of patients with sickle cell disease: A preliminary study

**DOI:** 10.3389/fmed.2023.1097466

**Published:** 2023-02-21

**Authors:** Khalid Ahmed, Yasamin Abdu, Sief Khasawneh, Ahmed Shukri, Ehab Adam, Salma Mustafa, Mohammad Affas, Mohamed Izham Mohamed Ibrahim, Abdullah Al Zayed, Mohamed A. Yassin

**Affiliations:** ^1^Department of Hematology, National Center for Cancer Care and Research (NCCCR), Hamad Medical Corporation (HMC), Doha, Qatar; ^2^Department of Community Medicine, Hamad Medical Corporation (HMC), Doha, Qatar; ^3^Department of Internal Medicine, Hamad Medical Corporation (HMC), Doha, Qatar; ^4^College of Pharmacy, QU Health, Qatar University, Doha, Qatar; ^5^Qatif Central Hospital, Qatif, Saudi Arabia

**Keywords:** sickle cell disease, intermittent fasting, vaso-occlusive crisis, hemolytic crisis, cbc

## Abstract

**Introduction:**

Sickle cell disease is a genetic disorder that frequently presents with vaso-occlusive crisis (VOC). Most patients with sickle cell disease in Qatar are Muslims; hence, they practice intermittent fasting during the holy month of Ramadan. However, there is a paucity of literature describing the effect of intermittent fasting on the occurrence of severe VOC. As a result, there is a lack of guidelines or standardized protocols that can help physicians advise patients with sickle cell disease who wish to practice intermittent fasting. Therefore, this study's aim was to investigate the effect of intermittent fasting on the clinical and hematological parameters of individuals with sickle cell disease.

**Methods:**

We conducted a retrospective study for 52 Muslim patients with sickle cell disease in Qatar aged ≥18 years who were confirmed to be fasting during the holy month of Ramadan during any of the years 2019–2021. The difference in the occurrence of severe VOC, hemolytic crisis, and other clinical, hematological, and metabolic parameters were studied one month before, during, and one month after the intermittent fasting of Ramadan using the patient's medical records. Mean (sd), median (IQR), and frequency (%) described the data. One-way with repeated measures ANOVA with a Greenhouse-Geisser correction and Friedman tests (^*^) were used at alpha level 0.05.

**Results:**

The study participants' (mean±sd) age was (31.1±9.2) years, 51.9% were males, and 48.1% were females. Roughly seventy percent of the participants were of Arab ethnicity, while the rest were either African or Asian. Most of the patients were homozygotes (SS) (90.4%). The median number of severe VOC (*P* = 0.7) and hemolytic crisis (*P* = 0.5) was not found to be significantly different before, during, or after Ramadan. Significant differences, however, were found in platelet count (*P* = 0.003), reticulocyte count (*P* < 0.001), and creatinine level (*P* = 0.038) with intermittent fasting.

**Discussion:**

In this preliminary study, intermittent fasting does not seem to influence the rate of occurrence of severe vaso-occlusive crisis or hemolytic crisis in patients with sickle cell disease; however, it was found to be associated with differences in platelet count, reticulocytes count, and creatinine level. The statistical and clinical significance of these findings needs to be confirmed in studies with a larger sample size.

## Introduction

Sickle cell disease (SCD) is a genetic disorder that results from a mutation in the beta-globin gene resulting in the replacement of the amino acid glutamic acid with valine at position number 6, Patients who are homozygous for the mutation have sickle cell anemia and represent the most prevalent and severe form of the disease. The disease can also result from compound heterozygote mutations such as S/C or S/β thalassemia with variable clinical expression (1). The most common complication of SCD is the vaso-occlusive crisis (VOC) which presents with severe body pain. SCD is also a systemic disease that can potentially involve all organs (2). Acute complications of SCD include infection, acute chest syndrome, priapism, stroke, splenic sequestration, hepatobiliary complications, and acute kidney injury (3). Chronic complications include avascular bone necrosis, pulmonary hypertension, heart failure, renal insufficiency requiring dialysis, retinopathy, and leg ulcers (3).

Intermittent fasting has recently gained much popularity as an effective method to reduce weight. It is now practiced by many people, including Muslims, during the holy month of Ramadan. There are different types of intermittent fasting; Ramadan fasting represents the 16/8 method (fasting for 16 h a day) (4). Calorie deprivation for some time can induce dynamic cellular changes, which can manifest in clinical changes. Since Ramadan fasting represents one of the five pillars of the Islamic faith, there is a solid public structure to support fasting during Ramadan. We observe an increase in the number of SCD patients visiting healthcare facilities during the holy month of Ramadan; however, there is not much literature available about the effect of intermittent fasting on developing SCD complications such as severe VOC; as a result, no guidelines or standardized protocols exist which can help physicians advise individuals with sickle cell disease who wish to practice intermittent fasting.

The prevalence of sickle cell disease in the Arabian gulf countries is reported in the literature from Qatar (3.9%), Bahrain (2.1%), Oman (3.8%), Yemen (0.95%), United Arab Emirates (1.9%), and Saudi Arabia (0.01–0.1%) (5–9).

This study investigated the effect of intermittent fasting during Ramadan on developing severe VOC and hemolytic crisis, as well as differences in the hematological and metabolic parameters of SCD patients before, during, and after intermittent fasting. Subsequently, the results of this study will enable our physicians to have some data upon which they can advise individuals with SCD about intermittent fasting. It will also lay a foundation for further studies addressing this topic. In the long term, the recommendations from this study and future similar studies can help reduce the healthcare cost that results from frequent admissions to the hospital and the utilization of laboratory services, medications, and other healthcare services to address sickle cell disease complications.

## Methods

After obtaining approval from the institutional research board (IRB), we retrospectively reviewed the medical records of 145 adult Muslim patients diagnosed with sickle cell disease, living in Qatar, and following up with the hematology department in a tertiary care center. Fifty-two patients were confirmed to be fasting during part or all of Ramadan for any of the years 2019–2021; the research team confirmed the fasting status through a telephone script. The participants fulfilled the inclusion criteria of being 18 years or older, Muslim, and residing in Qatar. We excluded patients younger than 18 years, non-Muslim, sickle cell trait, pregnant ladies, patients with established chronic kidney disease, or patients who were confirmed to have an infection, conditions with fluid loss that can potentially lead to dehydration (e.g., severe diarrhea, vomiting, polyuria, etc.) or any other apparent precipitating factor for VOC during fasting.

The following outcome variables before (within 1 month of Ramadan), during, and post-Ramadan (till 1 month after) were obtained by screening the electronic medical records of the study participants:

1- The number of severe VOC episodes (i.e., requiring visiting a healthcare facility) (VOC included painful crisis, acute chest syndrome, stroke, myocardial infarction, and priapism),
2- The number of hemolytic crises requiring visiting a healthcare facility,
3- The length of hospital stay,
4- The total dose of morphine used,
5- Requirements for antibiotics,
6- Requirement for exchange transfusion,
7- The requirement for ICU admission,
8- Number of days in ICU, if any,
9- The levels of Hemoglobin (Hb), white blood cells (WBCs), platelets (Plt), reticulocyte count, lactate dehydrogenase (LDH), bilirubin, urea, and creatinine.

Excel and SPSS program v 29 were used for data management and analysis. Mean (sd), median (IQR), and frequency (%) described the data. Inferential statistics were applied to determine the differences and associations between variables. One-way with repeated measures ANOVA with a Greenhouse-Geisser correction and Friedman tests (^*^) were used to measure the difference over time. A priori significance level was set at 0.05.

## Results

Table 1 below describes the patients' demographic characteristics and their fasting profiles. Patients' average age (mean) ± (SD) was (31.1) ± (9.2). Males were slightly more than females (51.9%). Most study participants were Arab (69.2%) and non-Qatari (63.6%). The homozygote form (SS) represented the most common type of SCD (90.4%). The average number of days fasted by the participants (mean) ± (SD) was 25.2 ± (6) indicating that the participants fasted most of the holy month of Ramadan. The (mean) ± (SD) for the severe vaso-occlusive crisis among the study participants in the year immediately preceding the intermittent fasting of Ramadan was 2.1 ± (1.2). The (mean) ± (SD) of Hemoglobin F among the study participants was (14.3%) ± (6.3%). Table 1 also describes whether the study participants received a top-up (simple PRBCs) transfusion 1 month prior to, during, or up to 1 month after Ramadan, most patients did not receive a transfusion in any of the three time periods.

**Table 1:** Patients' demographic, type of SCD, and fasting profiles.

**Item**		**Mean (SD)**	**Median (IQR)**	***n* (%)**
Age		31.1 (9.2)	31.0 (23.3–35.8)	
Gender	Male			27 (51.9)
	Female			25 (48.1)
Nationality	Qatari			19 (36.5)
	Non-qatari			33 (63.6)
Region	Arab			36 (69.2)
	African			10 (19.2)
	Asian			6 (11.5)
Type of SCD	SS			47 (90.4)
	SC			2 (3.8)
	SD			3 (5.8)
Number of fasting days		25.2 (6.0)	28.5 (22.0–30.0)	
Compliance with hydroxyurea during Ramadan^*^	Yes			20 (38.5)
	No			16 (30.8)
	Not applicable			16 (30.8)
Number of VOC up to 1 year before Ramadan		2.1 (1.2)	2 (1–3)	
Hemoglobin F level before Ramadan		14.3 (6.3)	13.2 (9.8–17.5)	
Simple PRBCs transfusion 1 month prior to Ramadan	Yes			2 (3.8)
	No			50 (96.2)
Simple PRBCs transfusion during Ramadan	Yes			2 (3.8)
	No			50 (96.2)
Simple PRBCs transfusion 1 month after Ramadan	Yes			1 (1.9)
	No			51 (98.1)

SCD, sickle cell disease; VOC, vaso-occlusive crisis; PRBCs, packed red blood cells; (^*^) note that the dose of hydroxyurea for the participants was the same as in the non-fasting state but the frequency is reduced to once daily during the holy month of Ramadan.

Table 2 illustrates the clinical and laboratory parameters among SCD patients 1 month before, during, and 1 month after Ramadan. One-way with repeated measures ANOVA and Friedman test indicated no significant difference in all the clinical parameters tested over time. One-way with repeated measures ANOVA with a Greenhouse-Geisser correction indicated significant differences for changes in platelet count (*P* = 0.003), reticulocytes count (<0.001), and creatinine level (*P* = 0.038) over time. Pairwise comparisons using the Bonferroni test indicated significant differences for the following: for platelet count, the significant difference was seen during intermittent fasting compared to post-intermittent fasting, with platelet count being lower during the fasting period; for reticulocyte count, the significant difference was seen when comparing the pre intermittent fasting values to the values during intermittent fasting as well as when comparing the values during intermittent fasting to the values in the post intermittent fasting period with reticulocytes count being lower during the fasting period, regarding creatinine the significant difference was found between the values in the fasting period compared to the post fasting period with creatinine being higher during Ramadan. With the consideration of covariates (age, gender, region, type of SCD, days of fasting, and compliance with hydroxyurea), there is a significant influence of region on the platelet count (*P* ≤ 0.001), none influenced the reticulocyte count (*P* ≥ 0.05) or creatinine level (*P* ≥ 0.05).

**Table 2:** Clinical and laboratory parameters among SCD patients 1 month before, during, and 1 month after Ramadan.

**Item**	**Normal range**		**1 month before**	**During**	**1 month after**	***p*-value**
Number of VOC		Mean (SD)	0.21 (0.41)	0.27 (0.45)	0.23 (0.43)	0.715
Number of hemolytic crises		Mean (SD)	0.08 (0.27)	0.13 (0.35)	0.13 (0.35)	0.535
Length of hospital stay (days)		Mean (SD)	1.12 (2.4)	1.37 (3.52)	0.88 (1.88)	0.623
The total dose of morphine (mg)		Mean (SD)	24.8 (59.0)	15.8 (55.0)	7.12 (16.96)	0.182
Use of antibiotics		Yes (*n*, %)	6 (11.5)	7 (13.5)	10 (19.2)	0.486^*^
		No (*n*, %)	46 (88.5)	45 (86.5)	42 (80.8)	
The need for exchange transfusion		Yes (*n*, %)	3 (5.8)	2 (3.8)	1 (1.9)	0.607^*^
		No (*n*, %)	49 (94.2)	50 (96.2)	51 (98.1)	
Admission to ICU		Yes (*n*, %)	3 (5.8)	2 (3.8)	1 (1.9)	0.607^*^
		No (*n*, %)	49 (94.2)	50 (96.2)	51 (98.1)	
Number of ICU days		Mean (SD)	0.13 (0.63)	0.12 (0.51)	0.00 (0.00)	0.596
WBC count × 10^3^/μL	4–10	Mean (SD)	8.62 (3.66)	7.75 (3.03)	9.07 (4.76)	0.212
Hb (g/dL)	13–17	Mean (SD)	9.86 (1.50)	9.74 (1.91)	9.97 (1.49)	0.761
Platelet count × 10^3^/μL	150–410	Mean (SD)	271.39 (138.92)	234.35 (104.40)	317.08 (158.90)	0.003
Reticulocytes count × 10^3^/ μL	50–100	Mean (SD)	198.84 (76.23)	150.34 (55.58)	197.36 (77.17)	< 0.001
Urea (mmol/L)	2.5–7.8	Mean (SD)	2.85 (1.31)	3.08 (0.98)	6.04 (10.95)	0.098
Creatinine (μmol/L)	62–106	Mean (SD)	65.69 (2.42)	67.90 (19.35)	60.38 (22.05)	0.038
Bilirubin (μmol/L)	0–21	Mean (SD)	33.89 (26.10)	25.27 (13.88)	31.87 (24.35)	0.063
LDH (U/L)	105–333	Mean (SD)	365.89 (150.12)	355.67 (153.83)	444.00 (136.03)	NA^**^

SCD, Sickle cell disease; VOC, Vaso-occlusive crisis; ICU, intensive care unit; WBCs, White blood cells; Hb, Hemoglobin; LDH, Lactate dehydrogenase; NA, not available; (^*^), One-way with repeated measures ANOVA with a Greenhouse-Geisser correction ± Friedman tests were used; (^**^), unavailability of LDH values for most of the cases in the three time periods did not allow for analysis of this variable.

## Discussion

This preliminary study intended to measure the effect of intermittent fasting on the clinical, metabolic, and hematological parameters of patients with sickle cell disease. Fasting is the voluntary abstinence from or reduction of some or all food, drink, or both (absolute) for a period typically between 12 h and 3 weeks, i.e., in a short-term, long-term/prolonged, or intermittent pattern (10). Intermittent fasting has many types, including the Eat-Stop-Eat diet, 24 h fast, 5:2 diet; fasting for 2 days per week, once or twice per week, alternate day fasting, the warrior diet; fasting during the day and eating a big meal at night, spontaneous meal skipping and the 16/8 method; fasting for 16 h each day (4). Ramadan fasting is primarily consistent with the 16/8 method; it is practiced by millions of Muslims all over the world for a whole lunar month (29–30 days) every year, and it involves abstaining from all types of food or drinks from dawn to sunset time (11). A unique difference between Ramadan fasting and the 16/8 method of intermittent fasting is that most Muslims take a meal 1–2 h before dawn, in this sense it allows for good hydration before starting the fasting. Calorie restriction during intermittent fasting can affect metabolic regulation, e.g., by altering circadian biology, the gut microbiome and modifiable lifestyle behavior and this, in turn, has been shown to have major public health benefits (4, 12). A systematic review by Stephanie et al. of 27 trials addressing weight loss in obese and overweight individuals (18 randomized controlled trials and nine trials that compared weight post intermittent fasting to baseline weight without a control group) found that studies with intermittent fasting reported a 0.8 to 13% weight loss compared to the baseline weight without serious adverse events; moreover, better glycemic control was seen in the studies that recruited patients with type 2 diabetes (13). In Germany, a one-year follow-up of 1,422 individuals on an intermittent fasting diet reported lower systolic and diastolic blood pressure in those who fasted for a longer time; the proposed mechanism for the reduction in blood pressure is increased parasympathetic activity, increased renal excretion of norepinephrine and improved sensitivity to insulin and natriuretic peptide (14). A systematic review and meta-analysis of six studies involving 417 patients with non-alcoholic fatty liver disease reported a significant difference in body weight, body mass index, and aspartate transaminase between the fasting and the control group and no significant differences in the triglyceride level and the total cholesterol (15).

The examples mentioned above illustrate the role intermittent fasting may play in the prevention and management of chronic diseases; nevertheless, no studies have investigated the role of intermittent fasting in patients with sickle cell disease. With sickle cell disease being one of the most prevalent genetic diseases worldwide and particularly in the middle east region, where most of the population are Muslims, it becomes essential to study the effect of intermittent fasting on the course of this chronic disease.

Our findings indicate that there is no statistically significant difference in the rate of occurrence of severe vaso-occlusive crisis or hemolytic crisis in individuals with sickle cell disease who practiced intermittent fasting. Our research, however, investigated only one type of intermittent fasting, which is the 16/8 method; further research needs to be directed toward sickle cell disease individuals who practice other types of intermittent fasting and increasing the sample size by recruiting more patients from neighboring middle east countries.

Our research indicates a significant difference in platelet count during intermittent fasting compared to post-intermittent fasting, with platelet count being lower during the fasting period, however, the mean did not fall in the thrombocytopenia range (< 150 × 10^3^/μL) and none of the study participants developed any bleeding during the fasting of Ramadan. Whether this reduction in platelets count with fasting could have a clinical significance on patients with sickle cell disease (e.g., would it affect the rate of occurrence of thrombotic events? Can intermittent fasting be used to lower platelet count in SCD patients?) or remains a statistical finding is not yet clear and more research is needed to confirm this finding and answer this question. Likewise, the reticulocyte count was significantly different during intermittent fasting compared to both before and post-intermittent fasting, indicating that the reticulocyte count acutely drops during fasting and quickly recovers when fasting is over. These two findings suggest that intermittent fasting can affect hematopoiesis; however, the exact mechanism is yet to be studied, and reduced calorie intake may be postulated as a cause for the slightly depressed platelet and reticulocyte count during intermittent fasting. Regarding kidney function, our research indicates that there is a significant difference in creatinine during intermittent fasting as compared to post-intermittent fasting; the repeated prolonged periods of reduced water intake may account for this, given that patients with sickle cell disease have varying degrees of sickle nephropathy which may render their kidneys sensitive to any periods of reduced perfusion. Interestingly an experimental study conducted in Senegal investigated the effect of Ramadan fasting on the hematocrit and blood viscosity by measuring these values in an experimental group of 10 patients with sickle cell trait and a control group of 10 patients without sickle cell trait during the fasting of Ramadan and 6 weeks after Ramadan. The measurements were done twice (at 8 AM and 6 PM) to account for intraday variations. While hematocrit did not differ between the two groups, it was greater in the evening compared to the morning regardless of the fasting status, the hematocrit mean was reported as (43 ± 1) in the morning for both groups compared to (45 ± 1) in the evening for both groups with *P* < 0.001. On the other hand, while no significant difference occurred in the blood viscosity for the control group throughout the day whether fasting or not, patients with sickle cell trait had a significant increase in blood viscosity in the evening during the fasting which might indicate a higher risk of impaired blood flow in the microcirculation (16).

## Limitations

- This is a pilot phase with a small sample size. Our plan is to conduct a multicentre study with a bigger sample size.
- We studied only one type of intermittent fasting; an extension of this study will try to investigate the other types.
- A case-control study with a control group of healthy individuals will be a better design to investigate the reduction of platelet count and the increase in creatinine during intermittent fasting.

## Conclusion

In this preliminary study, intermittent fasting during the holy month of Ramadan does not seem to influence the rate of occurrence of severe vaso-occlusive crisis or hemolytic crisis in patients with sickle cell disease; however, it can be associated with differences in platelet count, reticulocyte count, and creatinine level. Until stronger evidence is available from larger studies the decision to allow fasting for sickle cell disease patients should be carefully considered on a one-by-one basis considering the clinical characteristics of each patient. close follow-up from the treating physician is required with advice on when to break fasting. More studies with a bigger sample size are needed to confirm these results. Additionally, more studies are needed to look at types of intermittent fasting other than the 16/8 method.

## Data availability statement

The raw data supporting the conclusions of this article will be made available by the authors, without undue reservation.

## Ethics statement

The studies involving human participants were reviewed and approved by Institutional Review Board-Hamad Medical Corporation. The patients/participants provided their written informed consent to participate in this study.

## Author contributions

All authors listed have made a substantial, direct, and intellectual contribution to the work and approved it for publication.
